# Assessing mpox knowledge and sexual behaviours within high-risk populations in the Democratic Republic of the Congo

**DOI:** 10.1136/bmjgh-2025-019865

**Published:** 2026-05-18

**Authors:** Candice Lemaille, Megan Halbrook, Sydney Merritt, Yvon Anta, Lygie Lunyanga, Patrick K Mukadi, Emmanuel Hasivirwe Vakaniaki, Thierry Kalonji, Michel Kenye, Cris Kacita, Sylvie Linsuke, Isaac I Bogoch, Muge Cevik, Gregg S Gonsalves, Mikayla Hunter, Laurens Liesenborghs, Souradet Y Shaw, Robert L Shongo, Lisa E Hensley, Nicole A Hoff, Anne W Rimoin, Placide Mbala-Kingebeni, Jason Kindrachuk

**Affiliations:** 1 Department of Medical Microbiology and Infectious Diseases, University of Manitoba, Winnipeg, Manitoba, Canada; 2 Department of Epidemiology, University of California Los Angeles Jonathan and Karin Fielding School of Public Health, Los Angeles, California, USA; 3 Institut National de Recherche Biomédicale, Kinshasa, Congo (the Democratic Republic of the); 4 National Program for the Control of Mpox and Viral Hemorrhagic Fevers, Ministry of Health, Kinshasa, Congo (the Democratic Republic of the); 5 Department of Medicine, University of Toronto, Toronto, Ontario, Canada; 6 Division of Infection and Global Health, University of St Andrews, St Andrews, UK; 7 Department of Epidemiology of Microbial Diseases, Yale University Yale School of Public Health, New Haven, Connecticut, USA; 8 Public Health Modeling Unit, Yale University Yale Law School, New Haven, Connecticut, USA; 9 Department of Community Health Sciences, University of Manitoba Max Rady College of Medicine, Winnipeg, Manitoba, Canada; 10 Department of Clinical Sciences, Institute of Tropical Medicine, Antwerp, Belgium; 11 Zoonotic and Emerging Disease Research Unit, National Bio and Agro-Defense Facility, Agricultural Research Service, United States Department of Agriculture (USDA), Manhattan, Kansas, USA; 12 University of Kinshasa Medical School, Kinshasa, Democratic Republic of Congo; 13 South African National Bioinformatics Institute, University of the Western Cape, Cape Town, South Africa; 14 Department of Virology, Graduate School of Medicine, Osaka Metropolitan University, Osaka, Japan; 15 Department of Internal Medicine, University of Manitoba, Winnipeg, Manitoba, Canada; 16 Manitoba Centre for Proteomics and Systems Biology, Health Science Centre, Winnipeg, Manitoba, Canada

**Keywords:** Global Health, Infections, diseases, disorders, injuries, Community-based survey, Health Services Accessibility

## Abstract

**Background:**

Historically, the Democratic Republic of the Congo (DRC) has faced the greatest public health burden from mpox, including more than 70 000 probable cases from 1 January 2024 to 2 February 2025. However, there has been a relative paucity of investigation focused on mpox community engagement in DRC, including assessments of disease knowledge and risk perception.

**Methods:**

Given the ongoing Clade I mpox public health emergency of international concern, and the linkage between sustained human-to-human transmission and dense sexual networks, we sought to investigate mpox knowledge and sexual behaviours among key populations. Between 20 March 2024 and 25 August 2024, we recruited 2794 participants distributed across Kinshasa, Kwango and North Kivu provinces, with a focus in urban centres where mpox risk was considered high.

**Results:**

Most participants were considered other at-risk populations (948; 33.9%), followed by men who have sex with men (MSM, 828; 29.6%) and sex workers (897; 32.1%). Mpox knowledge, including transmission routes as well as sexual and health-seeking behaviours, was evaluated through questionnaires led by peer educators. Overall, only 6.1% of all participants reported prior mpox knowledge. Among this participant subset, zoonosis (‘direct contact with infected animals’) and ‘people living in high-risk areas’ were the most frequently selected options in regard to mpox transmission and populations at risk, respectively. When considering at-risk behaviours for mpox, those who identified as sex workers reported significantly higher risk sexual activities, including multiple sexual partners (80.3% of sex work participants), engaging in transactional sex (84.7%) and anonymous sex (80.8%) compared with MSM. However, both sex workers (44.8%) and MSM (56.7%) reported the highest health seeking behaviours for a suspected sexually transmitted infection.

**Conclusions:**

Our results highlight the need to evaluate knowledge in high-risk communities, especially in an endemic setting. Community engagement, which incorporates both mpox knowledge and risk perception activities, is inclusive of at-risk populations and is needed for ongoing mpox containment and mitigation efforts.

**WHAT IS ALREADY KNOWN ON THIS TOPIC**

- Since 2022, mpox has disproportionately affected some populations, such as people living in endemic countries, as well as those with high-risk activities, such as transactional sex. Community-based studies on mpox knowledge have been targeting healthcare workers or general members of the community, without separating sexual activities or orientation.

**WHAT THIS STUDY ADDS**

- This study focuses on the mpox knowledge of high-risk populations, which includes sex workers and men who have sex with men, in an endemic country, the Democratic Republic of the Congo (DRC).
- In an mpox-endemic setting such as the DRC, only 6.1% of at-risk populations reported prior mpox knowledge.

**HOW THIS STUDY MIGHT AFFECT RESEARCH, PRACTICE OR POLICY**

- Given the increased urban circulation of mpox and dearth of disease knowledge, these data have major implications for public health communications in the DRC.
- This finding underscores the need for adapted public health communication as continued human-to-human transmission is documented.

## Introduction

 In 2024, the WHO announced the second mpox public health emergency of international concern (PHEIC), 2 years following the first mpox PHEIC resulting from a global mpox outbreak that impacted over one hundred non-endemic countries.^1 2^ The Democratic Republic of the Congo (DRC), where Clade I monkeypox virus (MPXV; also referred to as mpox virus^3^) is endemic, has faced the largest public health burden from mpox globally. This includes more than 70 000 probable cases from 1 January 2024 to 2 February 2025.^4^ Historically, Clade I MPXV infections have been primarily driven through zoonotic contact with limited recorded secondary transmission events.^5^ However, the 2023 emergence of Clade Ib MPXV in South Kivu, DRC, has resulted in shifting clinical and epidemiological characteristics. This has included sustained patterns of human-to-human transmission associated with sexual or intimate contacts and concentration among sexual networks.^6 7^ Additionally, extensive APOBEC3 mutations have also been identified among MPXV genomes associated with sustained human-to-human transmissions, similar to that seen among Clade IIb MPXV^8^,^11^ Sustained Clade Ia mpox transmission and APOBEC3 mutations have also been identified in Kinshasa. As the capital of DRC, with a population of >17 million, identification in Kinshasa has further increased the complexity of the ongoing public health emergency and concerns regarding additional regional and/or international expansion of Clade I MPXV.^12 13^ The increasingly complex nature of MPXV transmission and association between sustained human transmission and dense populations, including sexual networks, has further broadened the definition of those populations considered at-risk for mpox. These at-risk populations (ARP) include those who self-identify as men who have sex with men (MSM), sex workers and people with frequent zoonotic contacts.^14^

There is a critical need for rapid deployment of mpox diagnostics and medical countermeasures while also enabling healthcare access for ARP in DRC.^15^ Moreover, given the international expansion of Clade Ib to neighbouring regions, including Burundi, Rwanda, Uganda and Kenya, there is a critical need for community engagement and mobilisation activities that are able to reach at-risk communities.^416^,^18^ This includes community-based research activities regarding vaccine uptake.

While vaccination is an effective way to protect the population, awareness about the disease is also a valuable tool. The evaluation of mpox knowledge and awareness has been limited within Central Africa, especially among MSM and sex workers. Studies have focused on healthcare workers or general members of the communities, without including specific behaviours or explicitly categorising key populations.^19^

Here, we conducted a cross-sectional survey to assess and describe mpox knowledge and sexual behaviours among MSM, sex workers and other ARP in urban centres across three provinces in the DRC: Kinshasa, Kwango and North Kivu. This investigation sought to assess mpox knowledge among populations at increased risk for mpox, including infection acquisition and transmission, while also evaluating high-risk sexual activities and health-seeking behaviours across these groups.

## Methods

### Recruitment and questionnaire administration

The sampling methods have been described in detail previously.^20^ In brief, between 20 March 2024 and 25 August 2024, we recruited 2794 participants over 18 years old, self-identifying as either MSM and/or sex workers, and a general at-risk community group reporting neither sex work nor MSM activity. The recruitment was done in three urban study sites: Kinshasa in Kinshasa province, Kenge in Kwango province and Goma in North Kivu province. Sex workers were identified among participants who indicated sex work as their primary or secondary occupation. Participants were categorised as MSM if they were classified as a man at birth and indicated ever having had sex with a male. MSM and sex worker networks were identified and accessed through facilities open to these communities or by peer educators who are connected to these communities. An additional ARP group was formed of participants who self-defined as non-sex workers and non-MSM and were recruited by peer educators or at selected recruitment sites such as bars/clubs, major migration hubs and health centres. ARP participants included but were not limited to hunters, healthcare workers, retailers, butchers or carpenters. Staff visited farms and abattoirs to enrol additional ARP members in close contact with animals–another route for possible mpox exposure. The recruitment was designed to obtain the same proportion of MSM and sex workers, as well as the same proportion of participants in each province.

The recruitment was conducted by peer-educators, who were enrolled to assist in selecting locations and times for recruitment and to initiate the sampling chain by approaching people in their community. At selected recruitment sites, participants were asked to complete in a tablet-assisted interview. The completed survey was stored on a secure server (SurveyCTO, V.2.0, Dobility). Questions included demographic information, sexual activities, occupation, animal-related activities and mpox knowledge. Key questions regarding mpox knowledge and risk behaviours can be found in online supplemental table 1. Interviews were conducted by health-trained professionals in French or the local language, including Swahili, Kikongo and Lingala. The deployed questionnaire was developed in collaboration with local staff and peer-educators in Kinshasa. Questions had been previously validated in past serosurvey work in DRC and those specifically pertaining to sexual risk factors were adapted from US-based instruments for cultural appropriateness.

**Table 1:** Demographic characteristics of participants

	All		MSM		Sex workers		MSM and sex workers		At-risk population	
	n	%	n	%	n	%	n	%	n	%
	2794	100.0	828	29.6	897	32.1	121	4.3	948	33.9
Sex										
Male	1519	54.4	831	100.0	4	0.5	118	100.0	566	54.7
Female	1275	45.6	0.0	0.0	806	99.5	0	0.0	469	45.3
Age										
Median (IQR)	27	(22–33)	26	(22–31)	26	(22–32)	27	(22–30)	29	(24–38)
18–24	1056	37.8	349	42.1	370	41.2	51	43.2	286	30.2
25–34	1125	40.3	355	42.9	387	43.1	51	43.2	332	35.0
35–49	552	19.8	115	13.9	136	15.2	19	16.1	282	29.7
50+	61	2.2	9	1.1	4	0.4	0	0.0	48	5.1
Education level										
Less than elementary school	254	9.1	54	6.5	130	14.5	0	0.0	70	7.4
Finished elementary	766	27.4	140	16.9	374	41.7	27	22.3	225	23.7
Graduated high school	1179	42.2	411	49.6	318	35.5	70	57.9	380	40.1
Apprentice	40	1.4	14	1.7	8	0.9	0	0.0	18	1.9
College or beyond	555	19.9	209	25.2	67	7.5	24	19.8	255	26.9
Study site										
Kinshasa, Kinshasa	939	33.6	222	26.8	299	33.3	107	88.4	311	32.8
Kenge, Kwango	965	34.5	319	38.5	312	34.8	9	7.4	325	34.3
Goma, North Kivu	890	31.9	290	34.7	286	31.9	5	4.1	312	32.9

MSM, men who have sex with men.

### Statistical approaches

Mpox knowledge was compared among MSM, sex workers, and the ARP, as well as across age groups and education levels. The three study sites were also compared against each other. Descriptive statistics were generated by calculating frequencies for demographic characteristics such as sex at birth, cohort type, age group, education level, and study sites. The χ² or, where appropriate, Fisher’s exact test, was used to determine the association of categorical variables for bivariate analyses.

Univariate analyses, specifically logistic regression models, were performed for variables with ORs and 95% CIs. A p<0.05 from the univariate analysis was used to determine variables included in the multivariate analyses. Potential confounders, such as age, education and province, were identified, and ORs were adjusted using multivariate analysis. No collinearity was found between the variables. Multivariate analyses included cohort type, age groups, education levels and study sites, and results were considered significant at p<0.05, with adjusted ORs (aOR) and 95% CIs.

Sexual behaviours were compared between our cohorts, which include MSM, sex workers and ARP. We used χ^2^ to compare the number of participants having a specific behaviour to the other groups. A p<0.05 was considered significant. All data analyses and figures were done using RStudio (R V.4.2.2).

## Results

### Demographic information

We recruited 2794 participants across three study sites in DRC, including 965 (34.5%) participants from Kenge, Kwango Province, 939 (33.6%) from Kinshasa, Kinshasa Province and 890 (31.9%) from Goma, North Kivu Province. Among all participants, 1519 (54.4%) identified as male and 1275 (45.6%) as female. The median age of participants was 27 years, with a total range between 18 and 86 years of age.

Among all participants, 897 (32.1%) self-identified as a sex worker as their primary or secondary occupation, with 893 (99.6%) of these respondents identified as female. Additionally, 828 (29.6%) of the participants self-identified as MSM. Of note, 121/2794 (4.3%) participants self-identified as both MSM and sex workers. We also assessed education levels among study participants: 19.9% reported their terminal education level as college or beyond, 42.2% as completing high school, 27.4% as completing elementary school and 9.1% as with less than an elementary school education (table 1).

### Knowledge of mpox

**Figure 1:**
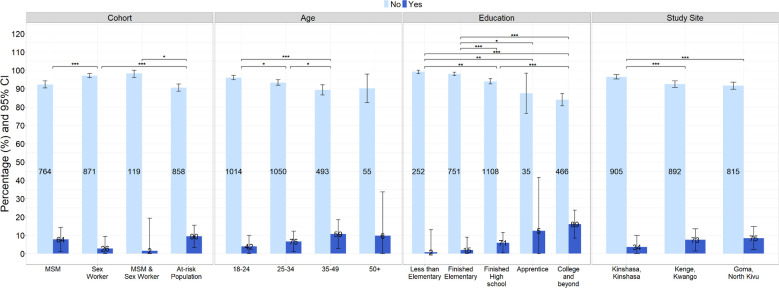
Answers to ‘Have you heard of the disease monkeypox or mpox?’ by different response groups. *p<0.05, **p<0.01, ***p<0.001. MSM, men who have sex with men.

Among all study participants, 6.1% (182/2794) had previously heard of mpox. This varied by population type: just 2.9% (26/897) of sex workers had heard of mpox the least of our population groups and significantly lower than both the MSM (7.7%, 64/828, p<0.001) and ARP populations (9.5%, 90/948, p<0.001). Among participants that identified as both MSM and sex worker, 1.7% (2/121) had previously heard of mpox, which was significantly lower than ARP (p<0.01). Mpox knowledge was significantly associated with age and education level (p<0.001, respectively). Here, 10.7% (59/552) of participants aged 35–49 years had heard of mpox as compared with 6.7% (75/1125) of those aged 25–34 years (p=0.03) and 4.0% (42/1056) of those 18–24 years (p<0.001). The proportion of participants aged 50 years and older who had heard of mpox (9.8%, 6/61) was not significant compared with the other age groups (p>0.05). Among those who reported less elementary school completion, 0.8% (2/254) of participants reported having heard of mpox as compared with 16.0% (89/555) of participants with college education or beyond (p<0.001). When considering geographic location, participants in Kinshasa reported less knowledge of mpox compared with those from Kenge (p<0.001) or Goma (p<0.001) (figure 1). Logistic regression models for age groups and education levels indicated that those aged 35–49 years (aOR: 2.81, 95% CI 1.79 to 4.45) or 50 years and older (aOR: 2.75, 95% CI 0.97 to 6.71) were more likely to have heard of mpox compared with younger participants aged 18–24 years. Moreover, participants with a college degree or higher were more likely to have heard of mpox as compared with those who had not completed elementary school (aOR: 0.04, 95% CI 0.007 to 0.14), participants who finished elementary school (aOR: 0.14, 95% CI 0.07 to 0.24) and those who graduated from high school (aOR: 0.40, 95% CI 0.28 to 0.56) (table 2).

**Table 2:** Odds of selecting an answer according to participants’ demographics

Variables	Knowledge about mpox			Knowledge about zoonotic transmission			Knowledge about human-to-human transmission			Visiting a healthcare facility if you suspect symptoms		
	n (%)	aOR	95% CI	n (%)	aOR	95% CI	n (%)	aOR	95% CI	n (%)	aOR	95% CI
Cohort												
MSM	64 (7.7)	0.88	(0.62 to 1.27)	56 (87.5)	1.22	(0.34 to 4.47)	47 (73.4)	0.74	(0.30 to 1.86)	470 (56.8)	1.07	(0.88 to 1.31)
Sex workers	26 (2.9)	0.54	(0.33 to 0.87)	25 (96.2)	0.63	(0.09 to 5.93)	21 (80.8)	1.06	(0.27 to 4.64)	389 (43.4)	0.70***	(0.57 to 0.86)
MSM and sex workers	2 (1.7)	0.28	(0.05 to 0.96)	2 (100.0)	--	--	0 (0.0)	--	--	96 (79.3)	1.68*	(1.0 to 2.78)
ARP	90 (9.5)	Ref	--	87 (96.7)	--	--	72 (80.0)	--	--	545 (57.5)	--	--
Age												
18–24	42 (4.0)	Ref	--	35 (83.3)	--	--	30 (71.4)	--	--	512 (48.5)	--	--
25–34	75 (6.7)	1.49	(1.00 to 2.25)	71 (94.7)	2.42	(0.73 to 8.46)	58 (77.3)	1.37	(0.50 to 3.76)	596 (53.0)	1.08	(0.90 to 1.30)
35–49	59 (10.7)	2.81***	(1.79 to 4.45)	58 (98.3)	3.03	(0.66 to 16.88)	46 (78.0)	1.1	(0.35 to 3.57)	351 (63.6)	1.54***	(1.22 to 1.94)
50+	6 (9.8)	2.75*	(0.97 to 6.71)	6 (100.0)	--	--	6 (100)	--	--	41 (67.2)	1.59	(0.90 to 2.90)
Education												
Less than elementary school	2 (0.8)	0.04***	(0.01 to 0.14)	2 (100.0)	--	--	1 (50.0)	--	--	108 (42.5)	0.42***	(0.3 to 0.58)
Finished elementary	15 (2.0)	0.14***	(0.07 to 0.24)	13 (86.7)	0.25	(0.03 to 2.21)	13 (86.7)	1.51	(0.30 to 11.66)	337 (44)	0.41***	(0.32 to 0.53)
Graduated high school	71 (6.0)	0.40***	(0.28 to 0.56)	68 (95.8)	0.46	(0.13 to 1.60)	53 (74.6)	0.71	(0.27 to 1.80)	681 (57.8)	0.71**	(0.57 to 0.89)
Apprentice	5 (12.5)	0.77	(0.25 to 1.90)	4 (80.0)	--	--	4 (80.0)	--	--	25 (62.5)	0.98	(0.50 to 1.97)
College or beyond	89 (16.0)	Ref	--	83 (93.3)	--	--	69 (77.5)	--	--	349 (62.9)	--	--
Study site												
Kinshasa	34 (3.6)	Ref	--	33 (97.1)	--	--	19 (55.9)	--	--	658 (70.1)	--	--
Kwango	73 (7.6)	2.01**	(1.30 to 3.16)	72 (98.6)	9.02*	(1.22 to 94.49)	60 (82.2)	5.38**	(1.74 to 17.68)	504 (52.2)	0.45***	(0.36 to 0.55)
North Kivu	75 (8.4)	2.21***	(1.43 to 3.50)	65 (86.7)	0.88	(0.22 to 3.04)	61 (81.3)	4.13**	(1.58 to 11.21)	338 (38.0)	0.25***	(0.20 to 0.31)

* p<0.05, **p<0.01, ***p<0.001.

aOR, adjusted OR; ARP, at-risk populations; MSM, men who have sex with men.

### MPXV transmission knowledge

Among the 182 participants who had previously heard of mpox, 93.4% (170/182) of participants selected at least one zoonotic mode of transmission provided in the questionnaire, whereas 76.9% (140/182) of participants selected human-to-human transmission. Participants who identified as both MSM and a sex worker (n=2), those aged 50 and older (n=6) and participants with less than an elementary degree (n=2) or apprentice (n=5) were excluded from the regression analysis due to insufficient sample size. When compared with each other, we did not find any statistical significance between responses selected from MSM, sex workers and ARP (p>0.05) (online supplemental tables 1 and 2).

We then assessed differences between mpox transmission knowledge and age, where the number of participants aged 18–24 years selecting a zoonotic transmission (35/42, 83.3%) was significantly lower than those aged 25–34 (p=0.008) or 35–49 (p<0.001). Our regression analysis demonstrated an association between knowledge of mpox transmission routes and age group—those aged 25–34 (aOR: 2.41, 95% CI 0.73 to 8.46) and 35–49 years (aOR: 3.02, 95% CI 0.66 to 16.88) were more likely to select a zoonotic route compared with those aged 18–24 (table 2).

Geographically, 74.0% (54/73) of the participants from Kenge selected three MPXV transmission modes compared with 53.2% (41/77) of participants from Goma and 44.1% (15/34) of those from Kinshasa. Among the transmission mode options provided, participants from Kenge (aOR: 5.38, 95% CI 1.74 to 17.68) and Goma (aOR: 4.13, 95% CI 1.58 to 11.22) were more likely to select ‘human-to-human transmission’ as compared with those from Kinshasa. Here, 55.9% (19/34) of participants from Kinshasa selected ‘human-to-human transmission’ compared with 88.2% (30/34) of Kenge’s participants (p=0.01) and 81.8% of participants from Goma (p=0.02). Moreover, the number of participants selecting a zoonotic mode of transmission was significantly less from Kinshasa residents compared with Goma (86.7%, 65/75, p<0.001) and those from Kenge (98.6%, 72/73, p<0.001).

### Health-seeking behaviours among participants

**Figure 2:**
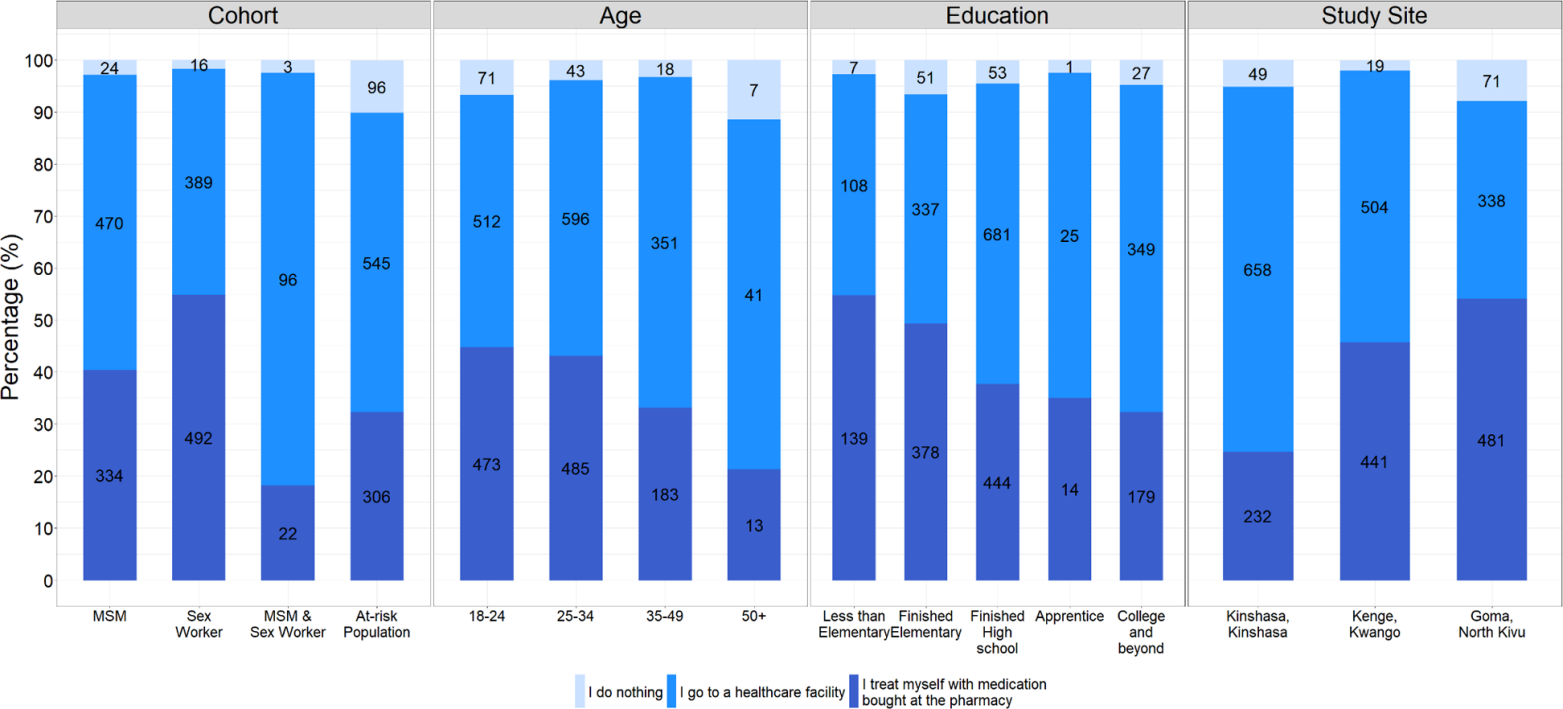
Percentage of participants for each course of action they would take in suspicion of an STI. MSM, men who have sex with men; STI, sexually transmitted infection.

Participants were also asked to select one action for the question: ‘Do you have any knowledge of how to recognise the signs of a sexually transmitted infection (STI) and what action to take in the event of a suspicion?’. Of the total population, 53.7% (1500/2794) selected ‘I go to a healthcare facility’, 41.3% (1154/2794) selected ‘I treat myself with medication bought at the pharmacy’, and only 5.0% (139/2794) of participants selected ‘I do nothing’ (figure 2; online supplemental table 3). More than half of the MSM (56.8%, 470/828) said they would go to a healthcare facility compared with 40.3% (334/828) of them saying they would treat themselves (p<0.001) or do nothing (2.9%, 24/8282, p<0.001). Among sex workers, we observed a similar trend, where 43.4% (389/897) would go to a healthcare facility, while 1.8% (16/897) would do nothing (p<0.001) and 54.8% (492/897) self-treat with medication from a pharmacy (p<0.001). As the age of the respondents increased, we observed an increase in reporting that they would seek care at a health facility (table 2). Participants aged 35–49 reported being more likely to seek healthcare from a professional compared with those aged 18–24 years (OR: 1.54, 95% CI 1.22 to 1.94). We observed similar trends by education level, where participants who completed high school (aOR: 0.71, 95% CI 0.57 to 0.89) had an elementary diploma (aOR: 0.41, 95% CI 0.32 to 0.53) and those with less than elementary school completion (aOR: 0.42, 95% CI 0.3 to 0.58) were less likely to go to a healthcare facility as compared with those with a college level or more.

Furthermore, the results showed the opposite for the participants who would self-treat with medication bought at the pharmacy (online supplemental table 3). Indeed, participants with lower education, as those with less than elementary (aOR: 2.28, 95% CI 1.64 to 3.18) and those with elementary (aOR: 2.08, 95% CI 1.61 to 2.68) or high school completion (aOR: 1.32, 95% CI 1.05 to 1.66) were more likely to select that health-seeking behaviour as compared with participants with college education. When looking at geographic location, participants from Kenge (aOR: 0.45, 95% CI 0.36 to 0.55) and Goma (aOR: 0.25, 95% CI 0.20 to 0.31) were less likely to seek healthcare from professionals, but more prone to treat themselves (aOR: 2.65, 95% CI 2.15 to 3.27 and aOR: 3.80, 95% CI 3.07 to 4.73, respectively), as compared with participants from Kinshasa.

### Assessment of sexual behaviours and mpox risk

**Figure 3:**
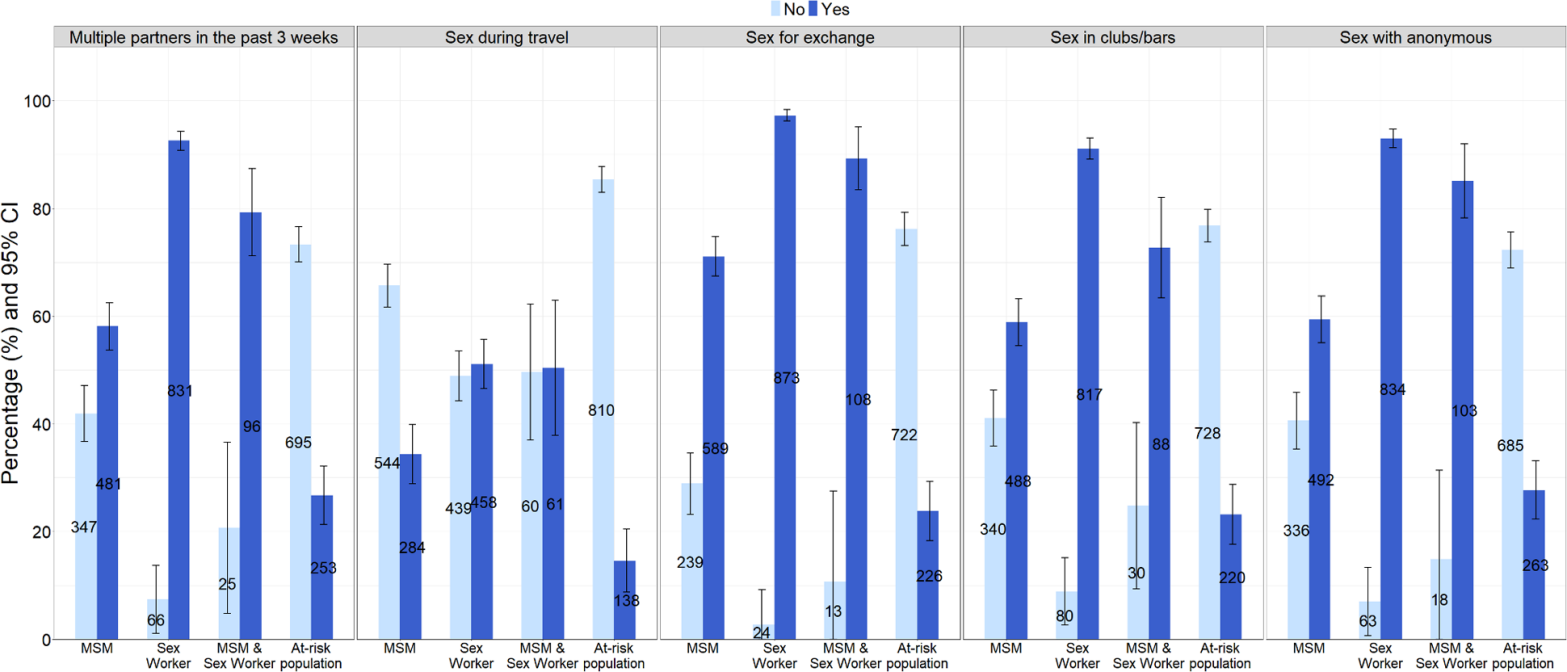
Percentage of groups of participants depending on their sexual activities. MSM, men who have sex with men.

We assessed sexual behaviours of the participants considered to increase mpox acquisition risk by evaluating the self-reported frequency of five different types of sexual activities (figure 3, online supplemental tables 4 and 5). In the overall population, 59.4% (1661/2794) of all the participants said they had sexual relations or intimate contacts with more than one person in the prior 3 weeks. This was significantly higher among sex workers as compared with MSM participants, with 92.6% (831/897) of sex workers reporting multiple partners in the prior 3 weeks compared with 58.1% (481/828) of MSM participants (p<0.001). Fewer respondents indicated having sex during travel, 33.7% (941/2794), as compared with other sexual activities provided in the questionnaire. Furthermore, 64.3% (1796/2794) of participants reported having transactional sex (including drugs, money, food or accommodations). When considering those who reported transactional sex participation among each group, a significantly higher percentage of those who identified as sex workers (97.3%, 873/897) answered yes as compared with those who identified as MSM (71.1%; 589/828, p<0.001). Among all the population recruited, 57.8% (1616/2794) of participants reported having sex in clubs or bars. Stratified by population type, 91.1% (817/897) of sex workers and 58.9% (488/828) of MSM participants reported sex in clubs or bars (p<0.001) (online supplemental table 3). Lastly, 1692/2794 (60.6%) participants said they ever had sex with anonymous partners, whereas most of the sex workers (93.0%, 834/897) reported this behaviour compared with MSM participants (59.4%, 492/828, p<0.001). A significantly greater proportion of participants who self-identified as both MSM and sex workers reported having sex in exchange for goods (89.3%, 108/121) as compared with MSM (p<0.001) or sex worker participants (p<0.001) (online supplemental table 4). This was also similar when considering those participants who self-identified as both MSM and sex workers reporting having anonymous sex (85.1%, 103/121) compared with MSM (p<0001) and sex workers (p=0.002).

## Discussion

Our results show a suboptimal level of awareness of mpox among populations at increased risk for mpox in the DRC, even in the context of the current outbreak. Indeed, only 6.1% of participants said they had previously heard of mpox, this included 2.9% of sex workers and 7.7% of MSM. Because only two participants who identified as both MSM and sex workers reported knowing about mpox, we did not account for this in our analyses. These gaps in mpox knowledge among key populations further emphasise the need for improved mpox communication–with targeted public health outreach strategies specific to these groups. We report increased mpox knowledge among participants aged 24 years and older, as well as those with college or greater education. The role of participant age in mpox knowledge can be linked to the participant’s employment: older participants were often clinicians and health professionals, while most younger participants worked in an unrelated health field or were currently unemployed.

By study site, mpox knowledge was more common among respondents in Kenge and Goma than in Kinshasa. This could be explained by the higher education level of Goma participants, and the older population distribution in Kenge. It should also be noted that mpox has not been known to circulate historically in Kinshasa, with subclade Ia mpox first reported in the city in August 2023 and subclade Ib introduction in July 2024.^12 13^ Given that the community engagement was made in a 5 month timeframe, it should be appreciated that the variation in mpox knowledge found between participants in Kinshasa and other study locations may differ following the broader circulation of mpox within Kinshasa.

Among the participants who had reported hearing of mpox before, we further assessed their knowledge of the modes of transmission and populations at the highest risk for mpox; the majority of MSM and sex workers were able to identify modes of transmission. For all sub-populations, the least selected mode of transmission was ‘human-to-human’. This is problematic since human-to-human transmission has been documented among sex workers in South Kivu, DRC in 2024,^21^ more globally among MSM during the clade IIb outbreak in 2022, and increasingly driving transmission in Kinshasa.^14^ Moreover, the percentage of participants from Kinshasa selecting ‘human-to-human transmission’ dropped to 55.9%, far less than the other study sites. This limited understanding of the transmission within the population needs to be addressed given the current co-circulation of subclade Ia and Ib in Kinshasa and reported sustained human-to-human transmission.^14 15^ When we looked at the answers to ‘Who is likely to contract mpox?’, the most selected response was ‘people living in high-risk areas’. This showed that people are aware of the risk in certain areas but do not necessarily realise that everyone can be at risk of contracting mpox. Our results in DRC contrast with mpox knowledge among Nigerian participants previously reported by Bakare *et al*.^22^ They reported mpox knowledge among 38.3% of their participants; however, it should be appreciated that participants were recruited in healthcare facilities. Moreover, the Bakare *et al* study demonstrated a good understanding of transmission routes and a high awareness about the perceived susceptibility of mpox among community members, with 58.8% of their participants aware that mpox can be transmitted during sexual intercourse. The contrasting results may demonstrate that considerable differences in mpox knowledge exist across geographic regions, even among endemic areas. This study was conducted during the 2022 global outbreak, where Nigeria accounted for 60% of the mpox cases in Africa, and human-to-human transmission has been sporadically reported since 2017. In our study, clade Ib was not circulating in some areas of the DRC, including two of our study sites, and transmission by clade Ia was primarily zoonotic. It is also appreciated that our study focused recruitment on populations at increased risk for MPXV infection at convenient social locations instead of healthcare facilities.

As mpox transmission is increasingly linked to sexual contact, we wanted to determine the sexual risk behaviours of high-risk populations, including key populations, in urban centres.^23^ The main objective of this study was to compare high levels of sexual behaviour to the mpox knowledge of our participants. Knowing that mpox has been circulating in dense sexual networks, we wanted to focus on MSM and sex workers as they are more at risk for exposure to the virus. Sex workers have occupational exposure since they use sex as a primary or secondary occupation. On the other side, MSM have more sexual behaviour, such as multiple partners, compared with ARP, which increases their risk of acquiring mpox. For each behaviour—sex in exchange for goods and services, multiple concurrent partners, etc—we observed higher reported sexual activity among sex workers, followed by MSM, as compared with ARP respondents. A large number of MSM reported having sex in exchange of goods; however, it could imply that they are the ones purchasing sex, which is different from sex workers who sell these services. Participants who self-identify as MSM and sex workers reported significantly more sexual behaviours, particularly anonymous sex and sex in exchange for goods, compared with MSM. The fact that they are also sex workers increases the number of behaviours since sexual activities are part of their occupation. However, while these participants showed to be at high risk for sexually transmitted diseases, the correlation between this and increased risk for mpox acquisition could not be determined. It raises concerns since these participants, with high levels of sexual activity, would also have a low level of mpox knowledge. The lowest reported risk behaviour for each population was having sex during travel. However, the question was only focused on sexual activities during travel and no question was asked about general travel frequency. This could bias our analysis since participants could have answered ‘no’ to this question only because they do not travel.

Finally, we sought to determine whether an individual would instead go to a healthcare facility, treat themselves or do nothing in the suspicion of an STI. The use of the health belief model allowed us to evaluate the perceived benefits of health actions. In our study, we assessed the perceived benefit of participants for seeking help in the suspicion of a sexual disease.^24^ The responses helped us understand if a participant was willing to seek help from a healthcare professional. This was related to understanding the behaviour of high-risk participants and whether they were open to receiving medication or vaccines as treatment or prevention for mpox. The health belief model has been used for HIV prevention to understand factors associated with condom use and what recommendations could be made to prevent AIDS.^25^ We showed that 59.8% of MSM and 49.2% of sex workers would consult a healthcare professional. Even with the stigma around their profession and/or their sexual activities, these groups are open to getting help from health professionals. This demonstrated that these individuals perceive the benefit of treating a sexual disease like STIs or mpox, by going to a healthcare facility, knowing they are at risk for stigma. The proportion of participants saying they would treat themselves decreased depending on their education level and age, where older people and those with higher education were more likely to go to a healthcare facility. This could be explained by the fact that older people had more knowledge and experience about STI and greater health-seeking knowledge. Similarly, people with higher education may be more informed about STIs, which correlates with the finding from Nigussie and Yosef, who demonstrated that the knowledge of STIs increases for each year of study.^26^ Furthermore, because 90% of healthcare is financed by private households in the DRC, older participants with higher education may have greater financial resources for treatment.^27^ However, this might confound their answer, as they would be less likely to seek care for minor symptoms or illness if they must pay for it.

When considering the study location, participants from Kenge and Goma were more likely to treat themselves with medication from a pharmacist as compared with participants from Kinshasa. Oleffe *et al* have shown that Congolese from Goma were more prone to purchase medicine at a pharmacy before seeking care from a healthcare professional due to better accessibility and lower costs.^28^ Furthermore, a recent report from the DRC Ministry of Health showed that 90.0% of Kwango residents went to a public setting to seek healthcare, while 48.8% of Kinshasa residents went to a private healthcare facility.^29^ While the perceived benefit of going to a healthcare facility was evaluated, questions about perceived barriers were not asked and would have been valuable to understand the different answers for the study cohort in our investigation.

Our study had limitations, which could affect the interpretation of our results. The sample size was focused on the different cohorts, ie, MSM, sex workers and ARP, and the study sites. Further, age group and education level were not considered in the recruitment process, leading to skewed distributions of age and education level attainment groups. Using peer-educators for recruitment could have led to selection bias, as they might have been more inclined to recruit friends or family members within their age range. The location, such as bars and clubs, could have reduced the recruitment of older participants who do not frequent these places. Moreover, for sexual activity-related questions, some questions had a time frame of 3 weeks or 6 months, followed by questions without a time frame which could have potentially impacted the interpretability by the participants. Lastly, the questions asked to each participant could have caused reporting bias, specifically social desirability bias. Indeed, sensitive questions about sexual behaviour or habits were asked, but some participants could have been reluctant to answer honestly.

In conclusion, this study highlights the important gaps in mpox knowledge among high-risk populations, high rates of high-risk sexual activities and limited understanding of mpox transmission routes. We demonstrated that the dearth of knowledge about mpox among key populations and frequent risk behaviours may contribute to the continued circulation of MPXV in urban centres, highlighting the need for targeted public health communication to at-risk cohorts. The help of peer-educators in the recruitment process showed that community involvement can be a considerable tool for outreach, and should be used more widely. While we call for the rapid deployment of materials to increase awareness in high-risk communities, further investigations are necessary to understand the accessibility of information and resources to protect them against diseases they can acquire in the course of their sexual activities.

## Supplementary material

10.1136/bmjgh-2025-019865 online supplemental file 1

10.1136/bmjgh-2025-019865 online supplemental file 2

## Data Availability

Data are available on reasonable request.
