# Supplementary material for: Assessing mpox knowledge and sexual behaviours within high-risk populations in the Democratic Republic of the Congo
Source: BMJ Glob Health. 2026 May 18;11(5):e019865. doi: 10.1136/bmjgh-2025-019865 (PMC13185043; doi:10.1136/bmjgh-2025-019865)
Supplement: online supplemental file 1 [file bmjgh-11-5-s001.docx]

**SUPPLEMENTAL MATERIAL - Assessing mpox knowledge and sexual behaviours within high-risk populations in the Democratic Republic of the Congo**

**Supplemental Table 1**: Mpox knowledge questions and the possible responses

| **Question** | **Responses** |
| --- | --- |
| Have you heard of the disease monkeypox or mpox? | Yes  No |
| What are the modes of transmission of the mpox virus? | Direct contact with infected animals  Consumption of meat from infected animals  Human-to-human transmission |
| Do you have any knowledge of how to recognize the signs of an STI and what action to take in the event of suspicion? | I treat myself with medication bought at the pharmacy  I go to a health-care facility  I do nothing |

**Supplementary Table 2:** Comparison of mpox-specific answers of MSM and sex workers according to the at-risk population (ARP)

| **Mpox-specific answers** | **Cohort** | **n** | **%** | **p-value** |
| --- | --- | --- | --- | --- |
|  |  |  |  |  |
| Zoonotic transmission | MSM | 56 | 87.5 | 1.00 |
|  | Sex Workers | 25 | 96.2 | 1.00 |
|  | ARP | 87 | 96.7 | Ref |
|  |  |  |  |  |
| Human-to-human transmission | MSM | 47 | 73.4 | 1.00 |
|  | Sex Workers | 21 | 80.8 | 1.00 |
|  | ARP | 72 | 80.0 | Ref |
|  |  |  |  |  |

**Supplementary Table 3**: Odds Ratios of selecting health-seeking behaviour according to the participants’ demographics

|  |  | **Visit to a healthcare facility** | | | **Self-treat with medication from the pharmacy** | | | **Do nothing** | | |
| --- | --- | --- | --- | --- | --- | --- | --- | --- | --- | --- |
| **Variables** |  | **n (%)** | **aOR** | **95% CI** | **n (%)** | **aOR** | **95% CI** | **n (%)** | **aOR** | **95% CI** |
| **Cohort** |  |  |  |  |  |  |  |  |  |  |
| MSM |  | 470 (56.7) | 1.07 | [0.88, 1.31] | 334 (40.4) | 1.33* | [1.08, 1.63] | 24 (2.9) | 0.24*** | [1.15, 0.38] |
| Sex Workers |  | 389 (44.8) | 0.70*** | [0.57, 0.86] | 492 (53.5) | 2.20*** | [1.79, 2.71] | 16 (1.7) | 0.12*** | [0.07, 0.21] |
| MSM & Sex Workers |  | 96 (80.5) | 1.68* | [1.05, 2.77] | 22 (16.9) | 0.86 | [0.51, 1.41] | 3 (2.5) | 0.20* | [0.05, 0.56] |
| ARP | Ref | 545 (55.2) | -- | -- | 306 (35.3) | -- | -- | 96 (9.5) | -- | -- |
|  |  |  |  |  |  |  |  |  |  |  |
| **Age** |  |  |  |  |  |  |  |  |  |  |
| 18-24 | Ref | 512 (48.5) | -- | -- | 473 (44.8) | -- | -- | 71 (6.7) | -- | -- |
| 25-34 |  | 596 (53.0) | 1.08 | [0.90, 1.30] | 485 (43.1) | 1.00 | [0.83, 1.20] | 43 (3.8) | 0.70 | [0.46, 1.06] |
| 35-49 |  | 351 (63.6) | 1.53*** | [1.22, 1.94] | 183 (33.2) | 0.77* | [0.61, 0.98] | 18 (3.3) | 0.43* | [0.24, 0.74] |
| 50+ |  | 41 (67.2) | 1.59 | [0.90, 2.90] | 13 (21.3) | 0.53* | [0.26, 1.00] | 7 (11.5) | 0.95 | [0.37, 2.15] |
|  |  |  |  |  |  |  |  |  |  |  |
| **Education** |  |  |  |  |  |  |  |  |  |  |
| Less than Elementary School |  | 108 (42.5) | 0.42*** | [0.3, 0.58] | 139 (54.7) | 2.28*** | [1.64, 3.18] | 7 (2.8) | 1.33 | [0.51, 3.08] |
| Finished Elementary |  | 337 (44.0) | 0.41*** | [0.32, 0.53] | 378 (49.3) | 2.08*** | [1.61, 2.68] | 51 (6.7) | 2.08 | [1.24, 2.54] |
| Graduated High School |  | 681 (57.8) | 0.71** | [0.57, 0.89] | 444 (37.7) | 1.32* | [1.05, 1.66] | 53 (4.5) | 1.35 | [0.82, 2.25] |
| Apprentice |  | 25 (62.5) | 0.98 | [0.50, 1.97] | 14 (35.0) | 1.07 | [0.52, 2.11] | 1 (2.5) | 0.77 | [0.04, 3.98] |
| College or beyond | Ref | 349 (62.9) | -- | -- | 179 (32.3) | -- | -- | 27 (4.9) | -- | -- |
|  |  |  |  |  |  |  |  |  |  |  |
| **Study Site** |  |  |  |  |  |  |  |  |  |  |
| Kinshasa | Ref | 658 (70.1) | -- | -- | 232 (24.7) | -- | -- | 49 (5.2) | -- | -- |
| Kwango |  | 504 (52.2) | 0.45*** | [0.36, 0.55] | 441 (45.7) | 2.64*** | [2.15, 3.27] | 19 (2.0) | 0.44** | [0.25, 0.76] |
| North Kivu |  | 338 (38.0) | 0.25*** | [0.20, 0.31] | 481 (54.0) | 3.80*** | [3.07, 4.73] | 71 (8.0) | 1.53 | [1.02, 2.31] |

*: p-value < 0.05

**: p-value < 0.01

***: p-value < 0.001

**Supplementary Table 4**: Comparison of sexual behavior of each group according to participant self-identified as MSM and Sex workers

| **Sexual behaviour** | **Cohort** | **n** | **%** | **p-value** |
| --- | --- | --- | --- | --- |
| Multiple partners in the prior three weeks  (n = 1661) | MSM | 481 | 58.1 | 0.00005 |
|  | Sex Workers | 831 | 92.6 | 0.000009 |
|  | MSM & Sex Worker | 96 | 79.3 | Ref |
|  | ARP | 253 | 26.7 | 1.72e-30 |
|  |  |  |  |  |
| Sex during travel  (n = 941) | MSM | 284 | 34.3 | 0.003 |
|  | Sex Workers | 458 | 51.1 | 1.00 |
|  | MSM & Sex Worker | 61 | 50.4 | Ref |
|  | ARP | 138 | 14.6 | 8.34e-21 |
|  |  |  |  |  |
| Sex in exchange for goods  (n = 1796) | MSM | 589 | 71.1 | 0.0001 |
|  | Sex Workers | 873 | 97.3 | 0.00005 |
|  | MSM & Sex Worker | 108 | 89.3 | Ref |
|  | ARP | 226 | 23.9 | 1.25e-47 |
|  |  |  |  |  |
| Sex in clubs/bars  (n = 1616) | MSM | 488 | 58.9 | 0.004 |
|  | Sex Workers | 817 | 91.1 | 7.74e-07 |
|  | MSM & Sex Worker | 88 | 72.7 | Ref |
|  | ARP | 220 | 23.2 | 1.15e-31 |
|  |  |  |  |  |
| Sex with anonymous  (n = 1692) | MSM | 492 | 59.4 | 2.84e-07 |
|  | Sex Workers | 834 | 93.0 | 0.02 |
|  | MSM & Sex Worker | 103 | 85.1 | Ref |
|  | ARP | 263 | 27.7 | 3.20e-35 |
|  |  |  |  |  |

**Supplementary Table 5**: Comparison of sexual behaviour between MSM and Sex workers

| **Sexual behaviour** | **MSM** | | **Sex Workers** | | **p-value** |
| --- | --- | --- | --- | --- | --- |
|  | **n** | **%** | **n** | **%** |  |
| Multiple partners in the prior three weeks | 481 | 58.1 | 831 | 92.6 | 1.46e-62 |
|  |  |  |  |  |  |
| Sex during travel | 284 | 34.3 | 458 | 51.1 | 1.29e-11 |
|  |  |  |  |  |  |
| Sex in exchange for goods | 589 | 71.1 | 873 | 97.3 | 7.44e-51 |
|  |  |  |  |  |  |
| Sex in clubs/bars | 488 | 58.9 | 817 | 91.1 | 1.10e-53 |
|  |  |  |  |  |  |
| Sex with anonymous | 492 | 59.4 | 834 | 93.0 | 1.78e-60 |
|  |  |  |  |  |  |
