# Supplementary material for: Assessing mpox knowledge and sexual behaviours within high-risk populations in the Democratic Republic of the Congo
Source: BMJ Glob Health. 2026 May 18;11(5):e019865. doi: 10.1136/bmjgh-2025-019865 (PMC13185043; doi:10.1136/bmjgh-2025-019865)
Supplement: online supplemental file 2 [file bmjgh-11-5-s002.docx]

### BMJ Global Health Author Reflexivity Statement

Adapted from Morton, B., Vercueil, A., Masekela, R., Heinz, E., Reimer, L., Saleh, S., Kalinga, C., Seekles, M., Biccard, B., Chakaya, J., Abimbola, S., Obasi, A. and Oriyo, N. (2022), Consensus statement on measures to promote equitable authorship in the publication of research from international partnerships. Anaesthesia, 77: 264-276. <https://doi.org/10.1111/anae.15597>

| **Study conceptualisation** | |
| --- | --- |
| 1. How does this study address local research and policy priorities? | This research directly addresses mpox risk perception among those communities that are at increased risk for mpox infection in DRC, including those that have stigmatized historically, following the expansion of mpox virus within the region due to sustained human-to-human transmission |
| 1. How were local researchers involved in study design? | Local researchers were involved in all aspects of the study design including acquisition of funding, identification of at-risk populations and recruitment of peer leaders, communication and messaging among communities, and interpretation of the survey results. |
| **Research management** | |
| 1. How has funding been used to support the local research team(s)? | Local researchers were included in the original funding applications (including as co-PIs), and funds were used to support field teams, research materials, and community engagement activities |
| **Data acquisition and analysis** | |
| 1. How are research staff who conducted data collection acknowledged? | All research staff were included as co-authors on this publication. |
| 1. How have members of the research partnership been provided with access to study data? | All co-authors have had access to the study data |
| 1. How were data used to develop analytical skills within the partnership? | Co-authors included teams from North America, DRC, and Europe and analytical skills were communicated and shared in all directions to ensure skill development from team members |
| **Data interpretation** | |
| 1. How have research partners collaborated in interpreting study data? | Research co-authors in DRC helped create the original survey instrument (and any question adaptations) as well as interpretation of answers were additional consultation was needed |
| **Drafting and revising for intellectual content** | |
| 1. How were research partners supported to develop writing skills? | All co-authors had access to open access platforms to facilitate co-development of the manuscript |
| 1. How will research products be shared to address local needs? | The direct connectivity of our research team, including our DRC co-authors, with the Ministry of Health will help ensure insights and knowledge from this work are considered in policy development and community engagement activities for ongoing mpox response activities, including vaccination campaigns |
| **Authorship** | |
| 1. How is the leadership, contribution and ownership of this work by LMIC researchers recognised within the authorship? | Leadership is recognized in our listing of corresponding authors and in ensuring that all local researchers were included in as co-authors |
| 1. How have early career researchers across the partnership been included within the authorship team? | Yes |
| 1. How has gender balance been addressed within the authorship? | Sex and gender balance has been considered where possible in our local research teams |
| **Training** | |
| 1. How has the project contributed to training of LMIC researchers? | This project is part of a large collaborative grant that was co-led by a DRC researcher. This grant and project have facilitated ongoing employment and engagement of local research staff at INRB in DRC. |
| **Infrastructure** | |
| 1. How has the project contributed to improvements in local infrastructure? | Where possible, infrastructure, including tablets, PPE, etc have been funded directly through collaborative grants for ongoing use by INRB in DRC. |
| **Governance** | |
| 1. What safeguarding procedures were used to protect local study participants and researchers? | Local field teams and researchers at INRB in DRC have extensive experience working across the country with community members. Where possible, recruitment for studies has been facilitated by peer leaders to ensure anonymity beyond communities |
